# Genome-Wide Association Study of Ustekinumab Response in Psoriasis

**DOI:** 10.3389/fimmu.2021.815121

**Published:** 2022-01-27

**Authors:** William T. Connell, Julie Hong, Wilson Liao

**Affiliations:** ^1^ Deparment of Pharmaceutical Chemistry, University of California San Francisco, San Francisco, CA, United States; ^2^ Insitute for Neurodegenerative Disease, University of California San Francisco, San Francisco, CA, United States; ^3^ Department of Dermatology, University of California San Francisco, San Francisco, CA, United States

**Keywords:** GWAS, psoriasis, ustekinumab, pharmacogenetics, precision medicine, pharmacogenomics

## Abstract

Heterogeneous genetic and environmental factors contribute to the psoriasis phenotype, resulting in a wide range of patient response to targeted therapies. Here, we investigate genetic factors associated with response to the IL-12/23 inhibitor ustekinumab in psoriasis. To date, only HLA-C*06:02 has been consistently reported to associate with ustekinumab response in psoriasis. Genome-wide association testing was performed on the continuous outcome of percent change in Psoriasis Area Severity Index (PASI) at 12 weeks of ustekinumab therapy relative to baseline. A total of 439 European ancestry individuals with psoriasis were included [mean age, 46.6 years; 277 men (63.1%)]. 310 (70.6%) of the participants comprised the discovery cohort and the remaining 129 (29.4%) individuals comprised the validation cohort. Chromosome 4 variant rs35569429 was significantly associated with ustekinumab response at 12 weeks at a genome-wide significant level in the discovery cohort and replicated in the validation cohort. Of psoriasis subjects with at least one copy of the deletion allele of rs35569429, 44% achieved PASI75 (75% improvement in PASI from baseline) at week 12 of ustekinumab treatment, while for subjects without the deletion allele, 75% achieved PASI75 at week 12. We found that differences in treatment response increased when rs35569429 was considered alongside HLA-C*06:02. Psoriasis patients with the deletion allele of rs35569429 who were HLA-C*06:02 negative had a PASI75 response rate of 35% at week 12, while those without the deletion allele who were HLA-C*06:02 positive had a PASI75 response rate of 82% at week 12. Through GWAS, we identified a novel SNP that is potentially associated with response to ustekinumab in psoriasis.

## Introduction

Psoriasis is a common, chronic immune-mediated skin disease that affects at least 2% of the population worldwide (1). Psoriasis is associated with psoriatic arthritis, cardiovascular disease, metabolic syndrome, and other comorbidities, which makes effective management of psoriasis critical. Moderate-to-severe psoriasis is treated with phototherapy and systemic agents, including targeted biologic inhibitors of TNF-*α*, IL-12/23, IL-17, and IL-23. Patient responses to biologic therapy can vary widely, from poor overall response to gradual loss of therapeutic sensitivity (2). Response differences are largely influenced by patient weight and adherence, drug dose and bioavailability, and pharmacokinetic covariates, such as drug immunogenicity (3). The molecular heterogeneity of psoriasis may also contribute to differential therapeutic responses. However, there are no molecular biomarkers routinely used in clinical practice to facilitate selection of the therapies tailored to individual patients.

Ustekinumab is a fully humanized immunoglobulin monoclonal antibody targeting the p40 subunit shared by IL-12 and IL-23. Phase 3 clinical trials showed that treatment with ustekinumab results in 75% improvement in the Psoriasis Area and Severity Index (PASI75) in ~66% of patients after 12 weeks of therapy (4–6). Candidate gene studies have identified the HLA-C*06:02 allele as being associated with better ustekinumab responses in both European (7–9) and Chinese (10) patients with psoriasis. A meta-analysis of eight studies including 1048 psoriasis patients showed that HLA-C*06:02 positive patients had a median PASI75 response rate of 92% after 6 months of ustekinumab therapy compared to a median PASI75 response rate of 67% in the HLA-C*06:02 negative patients (11).

Here, we performed an unbiased genome-wide association study (GWAS) to evaluate if additional genetic factors were associated with ustekinumab response. We evaluated our findings across multiple response timepoints and in conjunction with HLA-C*06:02. Our findings highlight a potentially novel variant associated with ustekinumab response in psoriasis, which may further facilitate the development of precision medicine approaches.

## Materials and Methods

### Study Population

This study involved analysis of individuals with moderate to severe psoriasis who participated in at least one of three placebo-controlled randomized clinical trials: PHOENIX I, PHOENIX II, and ACCEPT (4, 5, 12). Participants were originally approached for retrospective collection of DNA samples by investigators analyzing the association between the HLA-C*06:02 allele and response to IL-12/23 inhibition (7). In total, 439 patients of European descent were used to assess genetic associations between ustekinumab treatment and response.

**Figure 1:**
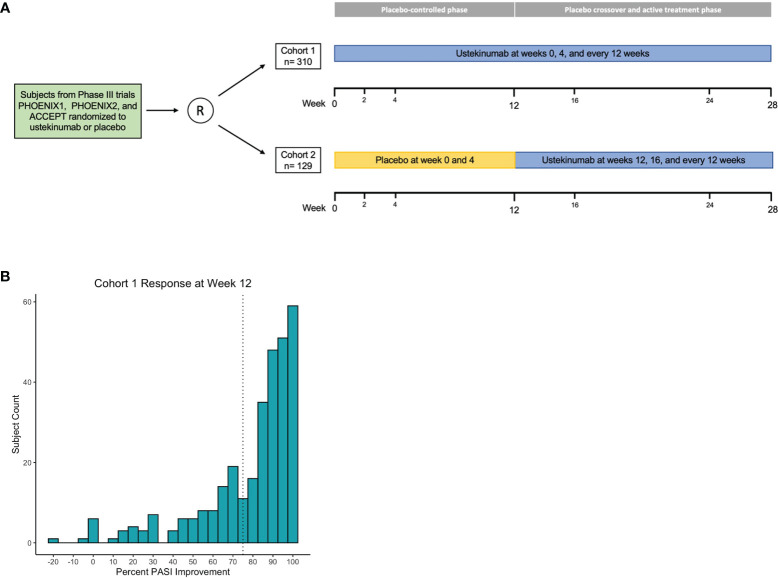
Association analysis design and primary outcome. Phase 3 clinical trial comprise discovery and validation cohorts **(A)**. Histogram of cohort 1 percent PASI improvement at week 12; dashed line marks 75% improvement threshold **(B)**.

The GWAS discovery cohort consisted of 310 individuals who were treated with 45mg (n=146) or 90mg (n=164) of ustekinumab for 40 weeks, with the lower or higher dose given according to body weight less than or greater than 100 kg, respectively. The validation cohort consisted of 129 trial participants who crossed-over from placebo to ustekinumab treatment at week 12 and continued ustekinumab for 16 weeks, again dose-stratified by body weight (45 mg: n=64; 90 mg: n=65). In both cohorts, ustekinumab was given with two loading doses 4 weeks apart and every 12 weeks thereafter (**Figure 1A**).

### Response Variables

In the ustekinumab phase 3 clinical trials, the primary endpoint was achievement of PASI75 at week 12. PASI75 is a binary outcome converted from percent PASI improvement from baseline. To maximize power for the GWAS, we focused on the continuous outcome measure of percent PASI improvement from baseline to 12 weeks after ustekinumab therapy. Phenotypic response to ustekinumab was recorded at weeks 2, 4, 12, 28, and 40 for the majority of patients in the discovery cohort (cohort 1). In order to validate findings, the placebo to ustekinumab cross-over patients acted as a validation cohort (cohort 2). PASI responses for cohort 2 were measured after 12 weeks of ustekinumab therapy compared to trial start.

### Genome Wide Association Study

Genotyping was performed using Illumina HumanOmni2.5-8 v1.2 BeadChips. Imputation was performed using the Michigan Imputation Server (https://imputationserver.sph.umich.edu/index.html) (13). The 1000 Genomes Phase 3 data was used as a reference panel for imputation (14). Files were converted to PLINK (v1.9) format, which along with R (v3.5.1) and python (v3.7.4), was used for data manipulation, visualization, and association analysis. Quality control and population stratification was performed following methods outlined by Marees et al. (15). Single nucleotide polymorphisms (SNPs) and individuals with missingness greater than 2% were removed. Duplicate, non-biallelic, and poor imputation quality (R^2^<0.7) SNPs were filtered. Non-autosomal SNPs with a low minor allele frequency (MAF<0.05) and significant deviation from Hardy-Weinberg equilibrium (*P*<1×10^-6^) were removed. In total 6,799,417 SNPs passed quality control, of which 1,696,820 were directly genotyped. Individuals with a heterozygosity rate +/-3 standard deviation from the mean were filtered, as well as the individual with the lowest call rate within a pair of cryptically related individuals $\left(\hat{\pi }>0.2\right)$ . In total, 310 individuals (181 males, 129 females) passed quality control. The previously described quality control steps were applied to the 1000 Genomes Phase 3 data prior to merging with cohort data for population stratification. Multidimensional scaling (MDS) was applied to the merged genotype information. The presence of ethnic outliers was evaluated by qualitative alignment with the European superpopulation cluster along the top 2 MDS components. We included the top 10 MDS components as covariates in linear regression models for association testing.

### Statistical Analysis

A threshold of *P*<5×10^-8^ was established in the discovery cohort to determine the associated markers for further replication. We took linkage-disequilibrium into account when interpreting multiple significant association results from the same region. Clumping was employed to greedily assign groups around index variants with *P*<5×10^-6^. Variants with an R^2^>0.5 and less than 1MB away were assigned representation by the index variant. We modeled the additive effect of allele dosage with the quantitative phenotype of interest using linear regression. When considering cohort 1 index variants in replication analyses, a 2-sided *t*-test with *P*<0.05 was considered statistically significant. A two-sided normal test for proportions (*P*<0.05) was applied to assess PASI threshold achievement differences based on genotype. The combined cohort association study followed the same procedures outlined for analysis of discovery cohort results.

### Power Analysis

We performed power calculations for the discovery and replication cohorts assuming an additive linear model for our quantitative trait of interest. Each power calculation was performed under consideration of the established type 1 error rates for the respective cohort (cohort 1 α, 5×10^-8^; cohort 2 α, 5×10^-2^). We examined power across a range of MAF (0.05-0.25) and effect sizes (ES) (1–9). The genpwr (v1.0.4) R package was used for all calculations.

## Results

In this study, we analyzed genetic data from two cohorts of psoriasis patients receiving ustekinumab. Following preprocessing and filtering for individuals of European genetic ancestry, the discovery cohort (cohort 1) totaled 310 individuals (181 males, 171 females) and the validation cohort (cohort 2) totaled 129 individuals (82 males, 47 females). The average PASI score at baseline was 18.6 for cohort 1 and 18.8 for cohort 2 (**Supplementary Table 1**). Power analysis revealed the discovery cohort had 1-β>0.75 for MAF>0.05 and ES>7. The replication cohort had 1-β>0.75 for MAF>0.05 and ES>5 (**Supplementary Figures 2A, B**). We used linear regression to perform genome-wide association testing on the percent improvement in PASI response at week 12 of ustekinumab therapy compared to baseline (**Figure 1B**). There was no correlation between age, BMI, and duration of the disease with the primary outcome of percent PASI improvement, and so these clinical variables were not included as covariates in the linear regression model (**Supplementary Figure 1**).

**Figure 2:**
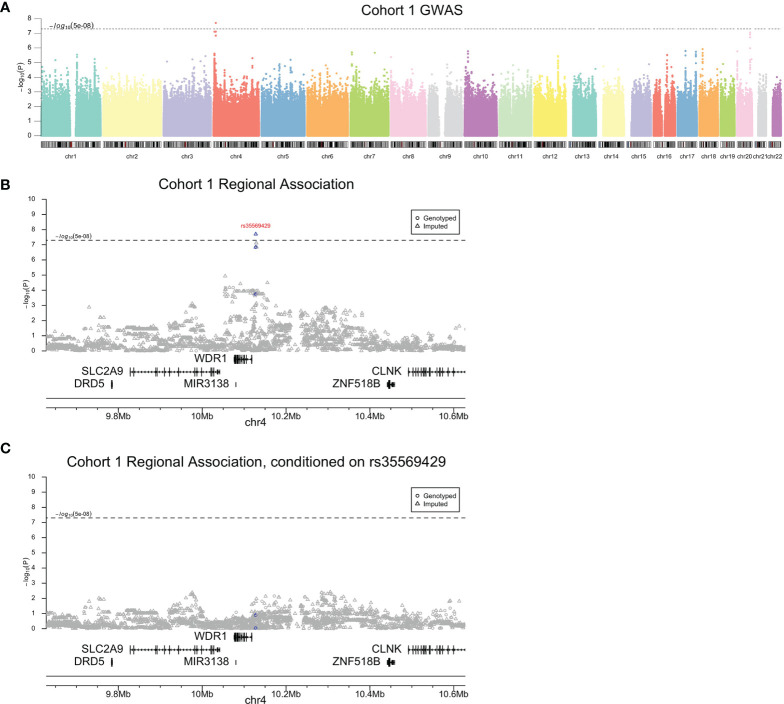
Cohort 1 association analysis results. Genome-wide **(A)**, regional **(B)**, and conditional association **(C)** Manhattan plots. Blue indicates variants in high linkage disequilibrium (R^2^>0.95) with rs35569429.

Genome-wide association testing of subjects in cohort 1 identified a single peak on chromosome 4 exceeding a genome-wide significance threshold of *P*<5×10^-8^ lead by rs35569429 (*β*, -19.84; 95% CI, -26.58 to -13.1; *P*=1.98×10^-8^) (**Figure 2A** and **Table 1**). Directly genotyped SNP rs11722643 was in high linkage disequilibrium with imputed SNP rs35569429 and achieved a similar level of significance (R^2^, 0.9; *β*, -19.31; 95% CI, -26.33 to -12.29; *P*=1.44×10^-7^). To determine whether multiple SNPs contributed to the peak on chromosome 4, we performed conditional analysis on rs35569429. The conditional analysis completely attenuated the GWAS peak, indicating a single independent signal at this locus (**Figures 2B, C**). The major allele of rs35569429 is “G” while the minor allele is a single nucleotide deletion of G, denoted as “Del”. Subjects with at least one minor allele were labeled as the deletion positive group (Del+, N=55), and subjects with zero minor alleles were labeled the deletion negative group (Del-, N=255). Only one subject was homozygous for the minor allele. To understand the impact of this SNP at various discrete levels of PASI response, we examined the proportions of Del- and Del+ individuals who achieved PASI50, PASI75, PASI90, and PASI100 at Week 12. We found that in the Del- group, 235/255 (92.2%) achieved PASI50, 191/255 (74.9%) achieved PASI75, 121/255 (47.5%) achieved PASI90, and 48/255 (18.8%) achieved PASI100 at Week 12. In the Del+ group, 39/55 (80.9%) achieved PASI50, 24/55 (43.6%) achieved PASI75, 12/55 (21.8%) achieved PASI90, 5/55 (9.1%) achieved PASI100 at Week 12.

**Table 1:** Cohort 1, 2 and combined association analysis results.

	SNP	MAF	β	*P* value
**Cohort 1**	rs35569429	0.090	-19.84	1.98E-08
**Cohort 2**	rs35569429	0.097	-6.71	0.042
**Cohort 1 + 2 Combined Analysis**	rs35569429	0.092	-15.83	2.42E-09

MAF, mean allele frequency.

**Figure 3:**
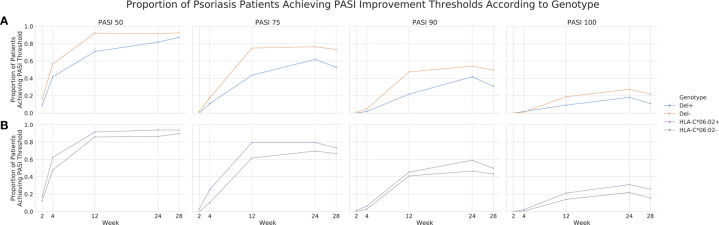
Proportion of psoriasis patients achieving PASI thresholds according to genotype in cohort 1. PASI 50, 75, 90 and 100 achievement across weeks 2, 4, 12, 24 and 28 for rs35569429 **(A)** and HLA-C*06:02 **(B)** genotypes.

To further investigate the validity of rs35569429, we analyzed its association with PASI outcomes in cohort 1 at timepoints that were not part of the original GWAS analysis (i.e. timepoints other than week 12). We found that a greater proportion of individuals in the Del- group achieved PASI75 compared to the Del+ group at Week 2 (1.57% *vs* 0%), Week 4 (17.6% *vs* 10.9%), Week 24 (76.5% *vs* 61.8%), and Week 28 (73.3% *vs* 52.7%) (**Figure 3A**). Similarly, the Del- group had a higher proportion of individuals achieving PASI50, PASI90, and PASI100 than the Del+ group at weeks 2, 4, 24, and 28. The difference in PASI responses between Del- and Del+ groups were generally comparable if not greater than the difference in PASI responses between HLA-C*06:02 positive and HLA-C*06:02 negative individuals (**Figure 3B**), where HLA-C*06:02 represents a previously well-validated locus associated with ustekinumab response (11). For comparison, in cohort 1, a linear regression of PASI response at week 12 for HLA-C*06:02, using 10 MDS components as co-variates, yielded β=0.7418 and *P*=0.0093.

We next investigated the association of rs35569429 with response to ustekinumab in an independent cohort 2. We found the same direction of effect at week 12 for rs35569429 (*β*, -6.71; 95% CI, -13.13 to -0.30; *P*=0.042) (**Table 1**). In the Del- group, 102/106 (94.5%) subjects achieved PASI50, 81/106 (76.4%) subjects achieved PASI75, 45/106 (42.5%) achieved PASI90, and 26/106 (24.5%) achieved PASI100 at Week 12. In the Del+ group, 20/23 (87.0%) subjects achieved PASI50, 13/23 (56.5%) achieved PASI75, 9/23 (39.1%) achieved PASI90, and 2/23 (8.7%) achieved PASI100 at Week 12. Association testing for rs35569429 in cohort 1 and cohort 2 combined at week 12 yielded a genome-wide significant result (*β*, -15.83; 95% CI, -20.72 to -10.74; *P*=2.42×10^-9^). We ran a sensitivity analysis on the full sample of cohorts 1 and 2 combined at week 12. We observed the expected genome-wide significant peak at rs35569429, with the most significant SNP being rs11722643, which is in high linkage disequilibrium with rs35569429 (R^2^, 0.88; *β*, -16.64; 95% CI, -22.04 to -11.25; *P*=3.25×10^-9^). We also observed a single additional genome-wide significant loci on chromosome 14, which could not be further confirmed (rs994384156; *β*, -14.94; 95% CI, -20.02 to -9.86; *P*=1.58×10^-8^). We also conducted a separate GWAS on ustekinumab response at week 24 and did not identify any genome-wide significant SNPs.

**Figure 4:**
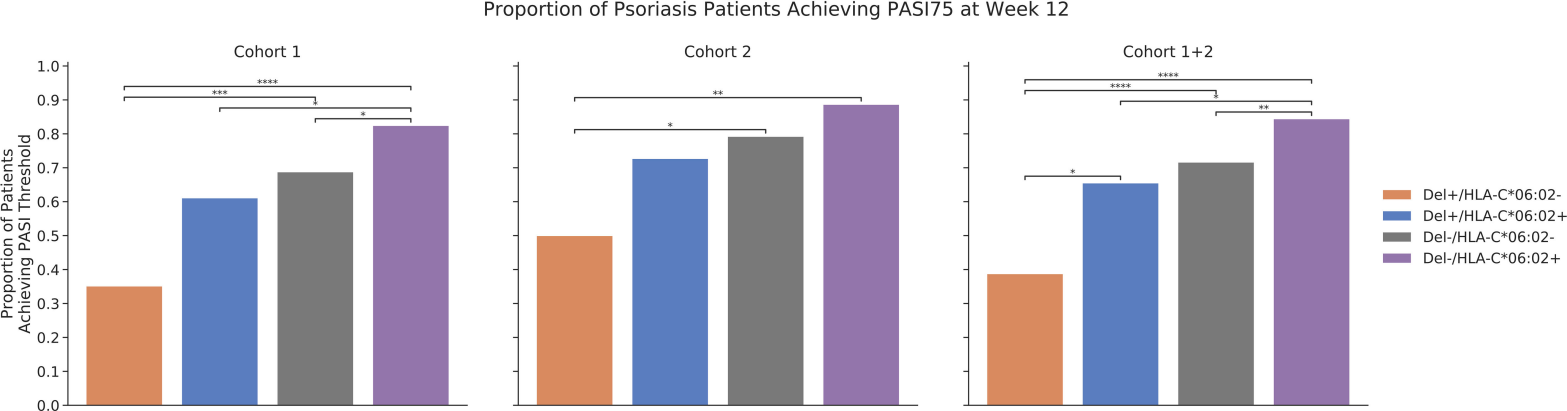
Proportion of psoriasis patients achieving PASI75 at week 12. *P<=5×10^-2^; **P<=1×10^-2^; ***P<=1×10^-3^; ****P<=1×10^-4^.

Finally, we explored how the combination of rs35569429 and HLA-C*06:02 affects PASI75 response in cohort 1 and 2 at week 12, since HLA-C*06:02 is an allele previously established to be associated with a more favorable responses to ustekinumab in psoriasis (11). In cohort 1 at week 12, 82.4% Del-/HLA-C*06:02+ individuals achieved PASI75 compared to 68.8% in Del-/HLA-C*06:02-, 61.1% in Del+/HLA-C*06:02+, and 35.1% in Del+/HLA-C*06:02- (**Figure 4**). In cohort 2 at week 12, 88.6% Del-/HLA-C*06:02+ individuals achieved PASI75 compared to 79.2% in Del-/HLA-C*06:02-, 72.7% in Del+/HLA-C*06:02+, and 50.0% in Del+/HLA-C*06:02-. In cohort 1 and cohort 2 combined at week 12, 84.4% Del-/HLA-C*06:02+ individuals achieved PASI75 compared to 71.6% in Del-/HLA-C*06:02-, 65.5% in Del+/HLA-C*06:02+, and 38.8% in Del+/HLA-C*06:02. The effects of rs35569429 and HLA-C*06:02 were independent from each other, as an interaction analysis that included an interaction term between rs35569429 and HLA-C*06:02 was not significant (*P*=0.729).

## Discussion

This genetic association study found a genome-wide significant association between intergenic variant rs35569429 and response to ustekinumab for the treatment of moderate to severe psoriasis. In our primary association analysis, absence of the minor allele (Del-) was significantly associated with a larger PASI improvement at 12 weeks from baseline. More favorable PASI responses in Del- individuals compared to Del+ individuals were also observed at weeks 2, 4, 24, and 28. The association of rs35569429 with ustekinumab response was validated in an independent cohort of psoriasis patients. Conditional analysis revealed a single independent signal at the locus of interest.

rs35569429 is characterized by a G deletion minor allele. This variant is located in an intergenic region 9 kB upstream of *WDR1*. Functional analysis by GeneHancer Regulatory Elements strongly associates a 10.6 kB region (GH04J010114) 1.2 kB downstream of this variant with promoter/enhancer activity influencing proximal protein coding genes *WDR1* and *SLC2A9* (16). The WDR1 protein is involved in actin filament disassembly, a critical process of cytoskeleton dynamics, especially in highly motile and interacting immune cells (17). Impaired actin dynamics as a result of WDR1 deficiency have been causally linked to primary immunodeficiencies and autoinflammatory phenotypes (18, 19). SLC2A9 is a transporter mainly expressed in the kidneys and primarily involved in urate reabsorption. Mutations of *SLC2A9* lead to poor reabsorption and Renal Hypouricemia type-2, as caused by increased urate excretion (20). Future studies are needed to fine-map the causal and functional SNPs in linkage disequilibrium with rs35569429.

Stratification of ustekinumab responses was greatest when rs35569429 was considered in combination with HLA-C*06:02. Individuals who were Del-/HLA-C*06:02+ achieved PASI75 84.4% of the time, while those were Del+/HLA-C*06:02- achieved PASI75 38.8% of the time, a more than two-fold difference.

Pharmacogenomics continues to play an increasingly important role in precision medicine for dermatology. In 2018, five dermatologic drugs had clinically actionable pharmacogenomic tags that either require or advise testing of genomic biomarkers before treatment (21). Single FDA-approved biomarkers currently dominate this list; however, multi-gene marker panels will continue to gain importance for informing clinical decisions. Understanding the role of multiple SNPs in disease pathogenesis is important in advancing precision medicine.

Conclusions from this study are limited due to the moderate sample size of the discovery and replication cohorts; our study was not powered for detection of small to moderate effects. Given the polygenicity of complex autoimmune diseases such as psoriasis, in the future, prospective design of large study cohorts is essential for thorough investigation of the biology contributing to therapeutic response. In general, validation in additional, independent cohorts will provide evidence with respect to the genomic signals discovered herein. Furthermore, the index SNP rs35569429 requires further investigation to identify the causal variant(s) associated with this locus and further characterization of functional effects on psoriatic response to ustekinumab.

## Data Availability Statement

The datasets presented in this study can be found in online repositories. The names of the repository/repositories and accession number(s) can be found below: (https://doi.org/10.6084/m9.figshare.17009930).

## Ethics Statement

The studies involving human participants were reviewed and approved by Institutional Review Boards of each clinical trial site participating in ustekinumab phase 3 studies. The patients/participants provided their written informed consent to participate in this study.

## Author Contributions

WL conceived and supervised the project. WC performed GWAS. WC and JH performed data analysis, prepared, and wrote the manuscript. All authors contributed to the article and approved the submitted version.

## Conflict of Interest

WL has received research grant funding from Abbvie, Amgen, Janssen, Leo, Novartis, Pfizer, Regeneron, and TRex Bio.

The remaining authors declare that the research was conducted in the absence of any commercial or financial relationships that could be construed as a potential conflict of interest.

## Publisher’s Note

All claims expressed in this article are solely those of the authors and do not necessarily represent those of their affiliated organizations, or those of the publisher, the editors and the reviewers. Any product that may be evaluated in this article, or claim that may be made by its manufacturer, is not guaranteed or endorsed by the publisher.
