# Supplementary material for: Genome-Wide Association Study of Ustekinumab Response in Psoriasis
Source: Front Immunol. 2022 Jan 27;12:815121. doi: 10.3389/fimmu.2021.815121 (PMC8830831; doi:10.3389/fimmu.2021.815121)
Supplement: Supplementary file 1 [file DataSheet_1.docx]

Supplementary Material

**Supplementary Table 1.** PASI Baseline. Average and standard deviation of baseline PASI for cohort 1, 2 and combined.

**Supplementary Table 1. Baseline PASI**

|  | Baseline avg week 0 | STD |
| --- | --- | --- |
| Cohort 1 | 18.6 | 6.32 |
| Cohort 2 | 18.8 | 6.62 |
| Cohort 1+2 | 18.6 | 6.40 |

**Supplementary Figure 1.** Correlation between cohort 1 clinical covariates and PASI improvement at week 12. Age (A), BMI (B) and disease duration (C) vs. percent PASI improvement at week 12. Formula and dashed annotation indicate line of best fit.

**Supplementary Figure 1A, 1B, 1C**


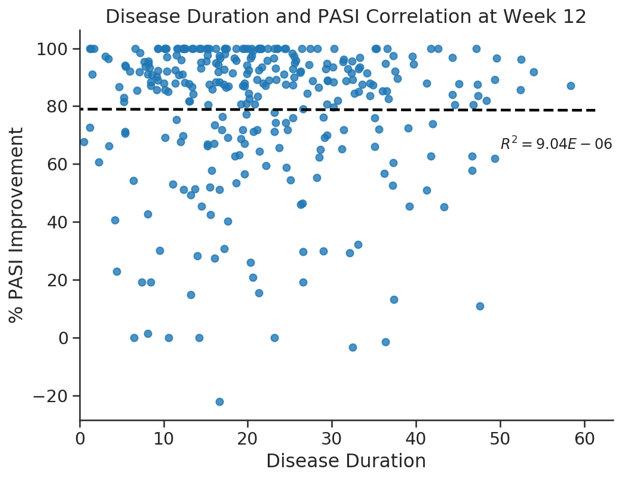

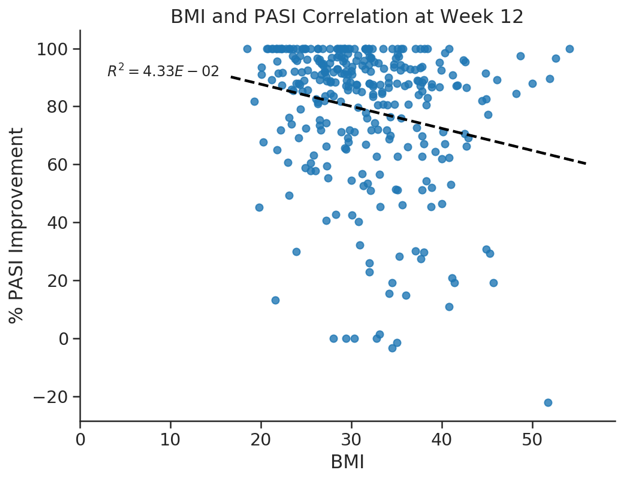

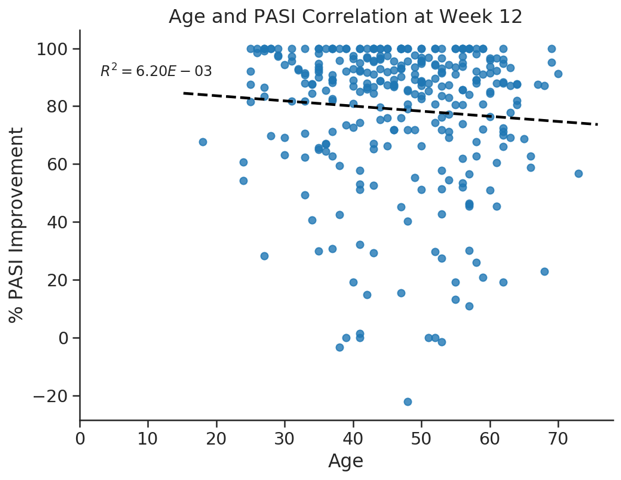


A

B

C

**Supplementary Figure 2.** Power analysis across a range of effect sizes (1-9) and minimum allele frequencies (0.05-0.25) for cohort 1 (A) and cohort 2 (B).

**Supplementary Figure 2A, 2B**


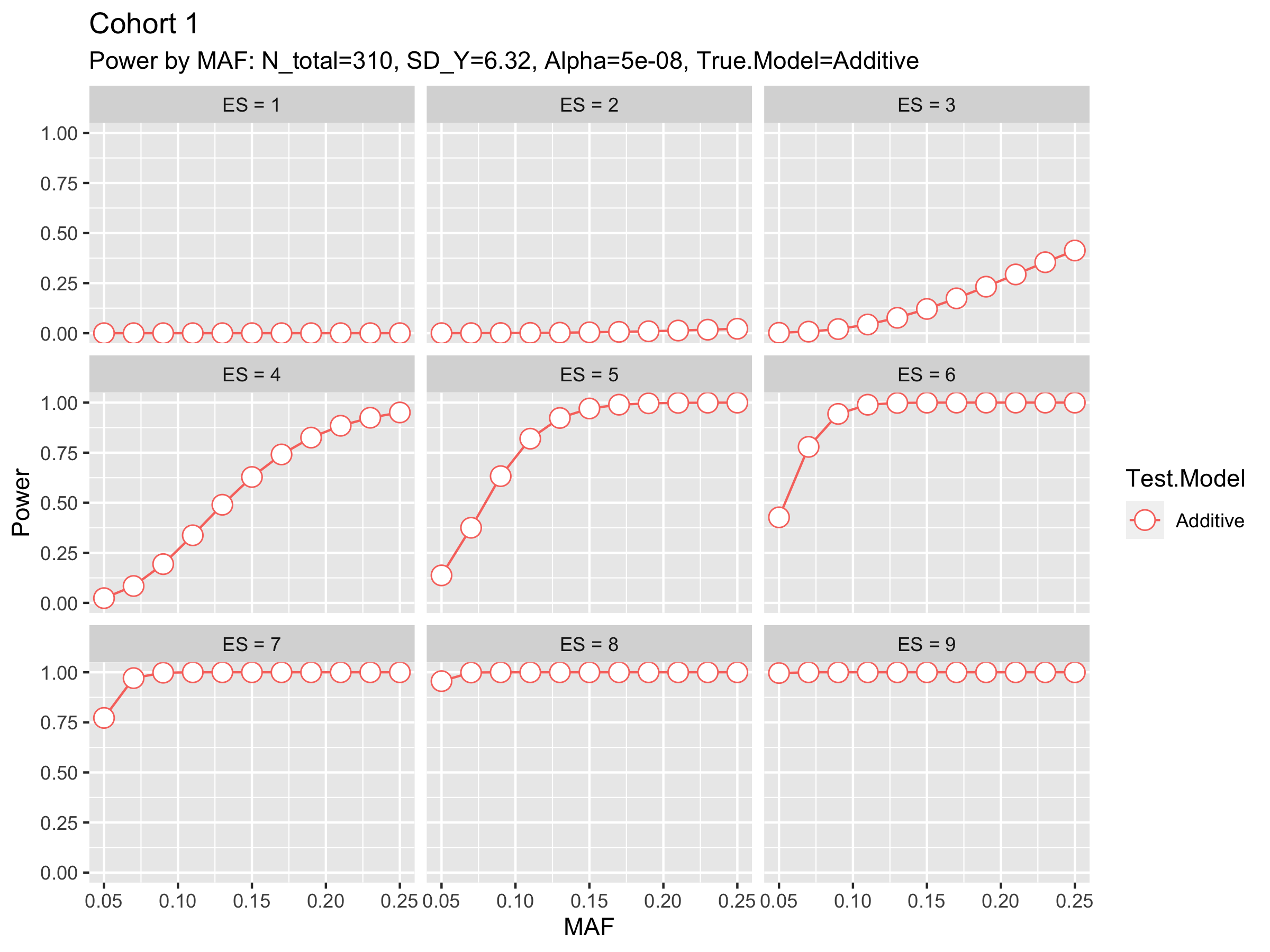

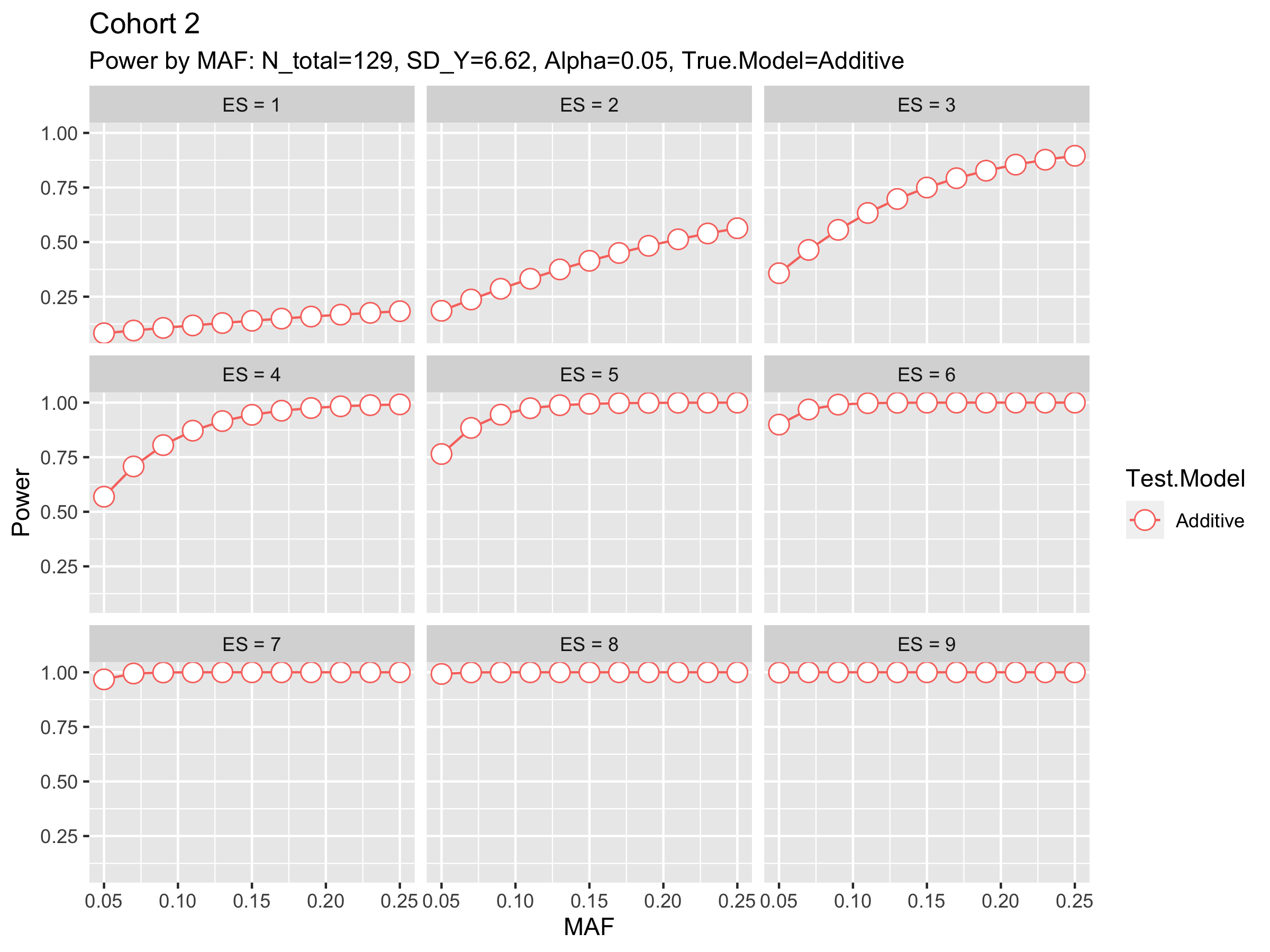


A

B
